# A typology of household-level adaptation to coastal flooding and its spatio-temporal patterns

**DOI:** 10.1186/2193-1801-3-466

**Published:** 2014-08-26

**Authors:** Jana Koerth, Athanasios T Vafeidis, Silvina Carretero, Horst Sterr, Jochen Hinkel

**Affiliations:** Coastal Risks and Sea-Level Rise Research Group, Institute of Geography, Christian-Albrechts-University Kiel, Ludewig-Meyn-Str.14, Kiel, 24098 Germany; Cátedra de Hidrología General, Facultad de Ciencias Naturales y Museo, Universidad Nacional de La Plata, Calle 64 n° 3, 1900 La Plata, Bs As, Argentina; Global Climate Forum e.V.(GCF), Neue Promenade 6, Berlin, 10178 Germany

**Keywords:** Accommodation, Types, Classification, Coastal flooding, Household-level adaptation

## Abstract

The predicted sea-level rise and changes in storm surge regimes are expected to lead to an increasing risk of flooding in coastal regions. Accommodation can be an alternative to protection in many areas, with household-level adaptation potentially constituting an important element of such a strategy, as it can significantly reduce costs. To date, a systematic typology of household-level adaptation to coastal flooding does not exist. In order to bridge this gap, we conducted a series of quantitative surveys in different coastal areas in Denmark, Germany and Argentina. We applied a cluster analysis in order to categorise the adaptive behaviour of coastal households. Coastal households were found to cluster in four groups that we term: the comprehensives, the theoreticians, the minimalists and the structurals. With the exception of households focusing on the implementation of high-effort structural measures, our results show the affiliation to these groups to follow a specific temporal sequence. At the same time, large differences in category affiliation exist between the study areas. Risk communication tools can utilise our typology to selectively target specific types of households or to ensure that the information needs of all groups are addressed.

## Introduction

The increased risk of flooding with rising sea levels (Nicholls and Cazenave 2010) and rapid population growth (Mc Granahan et al. 2007) indicates an urgent need for adaptation in coastal areas. It has been suggested that adaptation should be as low-regret (Wilby and Dessai 2010), flexible (Frankhauser and Soare 2013), and balanced in terms of costs and benefits (Hall et al. 2012) as possible. Nevertheless, it remains to be clarified how to achieve these targets in various regions of the world, especially in a proactive manner. Protection, as one possible type of coastal adaptation, is the most common approach of adapting to coastal hazards in many regions. During the 21st century it has shown to require substantial investments but also to be cost efficient for most developed coastlines (Hinkel et al. 2014; Nicholls 2009).

Accommodation can be an alternative to protection in many regions, particularly in those areas where protection is difficult to implement e.g. due to a complex coastal morphology, such as in deltas, or due to limited adaptive capacity in terms of technology and resources. When implementing accommodation measures, the use of land can be sustained, while existing structures and behaviours are modified. This can also involve the development of new structures, such as the construction of floating buildings or the modification of pre-existing structures to adapt to flood risk.

Of particular importance for accommodation strategies is the adaptation at the household level, as it can play a vital role in reducing the impacts of coastal flooding, mainly for the individuals themselves (Mendelsohn 2000; Tompkins and Eakin 2012). Integrating household-level adaptation into accommodation strategies also addresses the demand for assimilation of bottom-up stakeholder knowledge and top-down climate-impact projections (Mastrandrea et al. 2010). This would also be in accordance with the wider call for sharing the responsibility for adaptation (Adger et al. 2013).

Previous studies have explored household-level adaptation in coastal areas (e.g. Bichard and Kazmierczak 2011; Harvatt et al. 2011; Harwitasari and van Ast 2011; Molua 2012; Soane et al. 2010). It was shown that household-level adaptation to coastal hazards is widely applied by residents worldwide, despite being located in different regions and being surrounded by divergent institutional and social settings. The various measures range from every-day to high-cost actions.

Currently, however, there is no systematic grouping of adaptive behaviour of coastal households, nor is there knowledge on the measures different types of households are taking and how they differ between countries. This paper aims at bridging this gap in knowledge by developing a typology of households with regard to their adaptive behaviour. Such a typology can improve our understanding of which types of households are likely to implement which specific measures. This information can then be used for designing policy instruments, insurance schemes and risk communication tools that strategically target specific households.

For the purpose of categorisation, we conducted a household-level survey in coastal areas of Germany, Denmark and Argentina. The following four research questions are addressed in this study:

1. Which measures do coastal households take?
2. Can different categories of households applying similar combinations of adaptive measures be identified?
3. Can differences in allocation of household categories in the three countries be identified?
4. Can differences in behaviour be explained through differences in temporal characteristics?

The paper is organized as follows: Section 2 briefly reviews previous research on types of household-level adaptation. Section 3 describes the study area, the methodology applied and the sample. Section 4 presents the results. Section 5 discusses these results and section 6 concludes.

### Adaptation of coastal households

In this study, we define coastal anticipatory household-level adaptation as adjustments of individuals and households to expected future changes in sea-level rise related flooding, in order to decrease potential loss. Furthermore, household-level adaptation is understood as a type of accommodation, where existing structures and behaviours are modified.

Empirical studies on hazard mitigation/preparedness measures and household adaptation to coastal flood risk vary in the practices they investigate (e.g. Bichard and Kazmierczak 2011; Harvatt et al. 2011; Harwitasari and van Ast 2011; Koerth et al. 2013; Molua 2012; Soane et al. 2010; Terpstra 2011). Although these studies look at specific adaptation activities at the household level, a typology of households which implement combinations of specific measures is less frequently applied.

Due to the diversity of household adaptation, different classifications of adaptation measures have been constructed, but classifications of adaptive households are rare. Harvatt et al. (2011) categorized households into active and non-active types; non-active households respond with a do-nothing strategy as a result of denying the risk or living with it, whereas active households take reducing or changing measures. By classifying the measures undertaken by households, Thurston et al. (2008) categorize flood resistance and resilience measures into categories of temporary resistance (e.g. installing door guards), of permanent resistance (e.g. permanent flood proof external doors), of resilience without resilient flooring (e.g. raising electrics) and of resilience with resilient flooring (e.g. sealed floors). Linnekamp et al. (2011) categorize flood protection into individual actions, such as raising the level of own yards, and collective actions, such as assisting neighbours in undertaking protective measures. Classifications of adaptation measures and of households with regard to their adaptation behaviour also exist in studies on river flooding. Precautionary river flooding measures can be categorized according to their costs and efforts: low-cost measures (e.g. relocation of water-sensitive objects), medium-cost measures (e.g. flood-adopted interior fitting) and high-cost measures (e.g. flood proof air conditioning) are mentioned as household and household business measures by Kreibich et al. (2011). Adaptive behaviour can also be classified into protection against financial risks (by purchasing insurances), acquisition of information about precaution and precaution itself, by flood-proofing and retrofitting property (e.g. adapting use of building) (Thieken et al. 2007). An example of a classification of specifically structural protection measures is the one study carried out by the Department for Environment, Food and Rural Affairs (DEFRA London (2008)), which distinguishes between measures at property-level (e.g. against the entry of water into the house) and resilience (reduction of damaging by entered water). The classification methods applied in these studies differ; ad-hoc classifications are frequently used, whereas systematic approaches, such as statistical ones, have not been used extensively in the literature on household-level adaptation to coastal and river flooding.

### Study areas, data and methods

#### Study areas

The study areas are located in Germany, Denmark and Argentina. All areas have been affected by flooding in the past, though at different time periods, and are expected to experience a higher intensity and frequency of flooding in future. Coastal areas were selected on the basis of their physical exposure to sea-level rise. They are located in low-lying regions (elevation up to 5 m above mean sea-level and distance up to 5 km). The work was carried out in the context of the EU FP7 Comparative Assessment of Coastal Vulnerability to Sea-Level Rise at Continental Scale (COMPASS) project, which aims to analyse future impacts of sea-level rise and assess the vulnerability of coastal areas in South America and Europe.

In Germany, one meter of sea level-rise could potentially affect 300,000 people around the North Sea and the Baltic Sea. However, in this country, a high standard of public safety measures, mostly based on a hard protection strategy, exists in some regions, e.g. the North Sea coast (Sterr 2008).

Denmark has a high proportion (26%) of its land area located in the low elevation coastal zone (Mc Granahan et al. 2007). The west coast of Jutland, where the Danish study sites are located, is exposed to flooding and erosion. This is the only coastal area in Denmark, which receives financial support for protection from the government. In contrast to public responsibility in Germany, Danish coastal dwellers bear individual responsibility for protecting owned land, supported by project funding from the government (The Danish Government 2008).

The north-eastern coast of Buenos Aires Province in Argentina is affected by a regional typical storm called “sudestadas” (storm surges associated with high-energy waves due to strong wind), which causes coastal erosion and flooding (Pousa et al. 2007). The coastal area is characterized as being highly vulnerable to sea-level rise (Diez et al. 2007). Barragán Muñoz et al. (2003) criticize that there is a lack of specific policies in Argentina and that a central institutional organization is responsible for the coastal management.

Existing national policy frameworks on adaptation to climate change impacts and, specifically, to coastal flooding, differ between the study sites. The national adaptation strategies of Denmark and Germany seem to be similar: In both countries, for example, coastal risk management is considered a key issue addressed in the National Adaptation Strategies (Biesbroek et al. 2010). A large part of the German North Sea coast is protected by dikes; flood control and coastal protection are the responsibility of the state (Federal Ministry for the Environment, Nature Conversation and Nuclear Safety Germany 2009). In Denmark, landowners are responsible for protecting their properties from flooding (The Danish Government 2008). In Argentina, there is a lack of institutional organization responsible for the coastal management. An exception to this is the province of Buenos Aires, where the “Unidad de Coordinación de Manejo Costero Integrado” (Coordination Unit of Integrated Coastal Management) was created in 2008. However, there is no specific reference to flood management or adaptation strategies. Along the Argentinean coast there are a few examples of coastal protection structures and practices, such as seawalls, breakwaters, artificial beach nourishment and dune maintenance.

Beyond the policy context, differences in the socio-economic characteristics of households between the study sites may also lead to differences in adaptive behaviour. The Human Development Report shows that the overall level of development in Argentina, Germany and Denmark is characterized as being very high (Malik 2013). Still, significant differences between the three countries exist. Germany has the fourth highest gross domestic product (GDP) in the world (The World Bank 2014b). Denmark is characterized by low income inequality (Ortiz and Cummins, 2011) and high life satisfaction (Malik 2013). Argentina also has a comparatively high and increasing GDP (The World Bank 2014a) and inequality has decreased in recent years (Ortiz and Cummins, 2011).

### Questionnaire and survey design

A quantitative survey was carried out for each study area. Questionnaires were distributed in eight localities, using a stratified random sampling scheme. The respondents were asked to complete the questionnaires and return them by prepaid post. Interviewer and social desirability biases are known to be reduced with this methodology; however, biases caused by an inability to provide clarifications or due to a lack of interest of potential respondents can occur and need to be taken into account. Originally, the questionnaire was drafted in English and later translated into Danish, German and Spanish.

The questionnaire begins with a short paragraph setting the context of the survey by introducing the topic of flooding due to climate-induced sea-level rise and the inherent uncertainties in forecasting the extent of changes. Adaptation is explicitly mentioned with regard to changes linked to climate change. The explanations are short and avoid technical jargon and complex terms. The questionnaire then proceeds with questions on 29 adaptation measures, with responses on a binary scale, in order to obtain information related to their implementation. Since the questionnaire asks for both past and current behaviour, we assume that the possible bias, introduced by the preliminary explanations, to the replies in the second part of the questionnaire is negligible. The list of adaptation measures and their indicators was developed based on a literature review of measures and variables used in previous studies (Bichard and Kazmierczak 2011; Harvatt et al. 2011; Harwitasari and van Ast 2011; Molua 2012; Soane et al. 2010) as well as on expert knowledge. Finally, the questionnaire includes questions on a number of demographic, housing and other variables such as age, gender, occupancy, personal experience and reliance on public measures.

## Methods

The analysis was carried out in two steps. First, we employed a cluster analysis in order to investigate whether we can identify categories of households likely to carry out a number of similar adaptation measures at the same time. Cluster analysis is a form of statistical analysis, which aims at classifying objects systematically. Cases or variables, which can be characterized by a set of solid values, are grouped into clusters according to their similarity. These clusters should be as homogeneous as possible internally and as heterogeneous as possible externally. We thus clustered the households that applied similar adaptation measures. In order to avoid chaining and outliers, we used the squared Euclidean distance as a proximity measure and the ward as a clustering algorithm (Kaufmann and Rousseeuw, 2005). To test the stability of results, different methods employing varying proximity measures and clustering algorithms were applied. As variables were binary, they were not standardized. Data records with missing values were excluded; thus, 75.1% of respondents were finally selected and grouped.

In a second step, we conducted uni- and bivariate statistical analysis and Χ^2^ as well as Eta tests to analyse, whether the clusters attained (i) differ in their distribution between the sample sites, indicating that membership to a specific household type depends on the historical or institutional context; and (ii) could be explained through temporal variables on which data was also collected through the questionnaire, i.e. age of the respondent as well as length of residence in the house and at the coast. We hypothesise the household-level adaptation not to differ much spatially, as the exposure of coastal dwellers in the three study regions is similar. We further hypothesise the households to begin by implementing low-cost measures, e.g. reading information leaflets, and to only then conduct measures that involve higher expenditures, e.g. buying protective barriers for the doors of the house. This would imply temporal differences between type memberships.

## Results

### The sample

1459 questionnaires were handed out during the storm surge seasons in 2010 and 2011. 361 questionnaires were returned (a response rate of 24.74%), 35.7% were from Denmark, 35.5% were from Germany and 28.8% were from Argentina. A profile of the sample regarding demographic and housing data is given in Table 1. Age distribution is negatively skewed; nearly half (45%) of the respondents were older than 60 years. Most of them were male (58%) and 27% had children under the age of 18 years living at home.

**Table 1:** **Demographic and housing variables**

Variable	Values		Percentage
Age	< 20 years		0.9
	21 – 30 years		5.8
	31 – 40 years		9.3
	41 – 50 years		16.2
	51 – 60 years		23.2
	61 – 70 years		29
	> 71 years		15.7
Gender	Female		42.6
	Male		57.4
Children (up to 18 years) in same house/flat	Yes		26.7
	No		73.3
Ownership	Tenant		17.2
	Owner		82.8
Flooding damage(of house/flat/lot) in the past	Yes		30.7
	No		59.6
	Do not know		9.7
Knowledge about height above sea-level*	Yes		50.3
	No		49.7
Knowledge about distance to coastline*	Yes		86.3
	No		13.7
Existence of public safety measures	Yes		68
	No		28.8
	Do not know		3.2
Knowledge of height of public safety measure*	Yes		55.5
	No		44.5
**Demographic and housing**	**M**	**SD**	**min – max**
Occupancy house/flat in years	18.2	15.5	0.5 - 86.0
Occupancy coast in years	34.1	20.9	0.5 - 86.0
Height about sea-level in m**	3.9	2.9	-3 - 10
Distance to coast in m***	556	1173	3 - 5000

Note: *Self-reported, **Excluding values above 10 m, ***Excluding values above 5000 m.

The majority of respondents owned their house or flat (83%). This disproportional distribution possibly reflects the stronger concern of home owners regarding household-level adaptation compared to tenants (e.g. to reduce potential damage to the property) and therefore their stronger interest in participating in such a study. 31% of houses or flats have been damaged by flooding in the past. 50% of respondents indicated that they know the elevation above sea-level of their house/flat and 86% stated that they know the distance between house/flat and coastline. Average elevation above sea-level was estimated at 3.9 m (excluding outliers of locations above 10 m) and the average distance to the coast was estimated by respondents at 556 m (excluding locations beyond 5000 m). 68% mentioned the existence of public safety measures (e.g. dike) between their house/flat and the coastline. Average occupancy was 18.2 years in the current house/flat and 34.1 years at the coast in general.

### Which measures do coastal households take?

**Figure 1:**
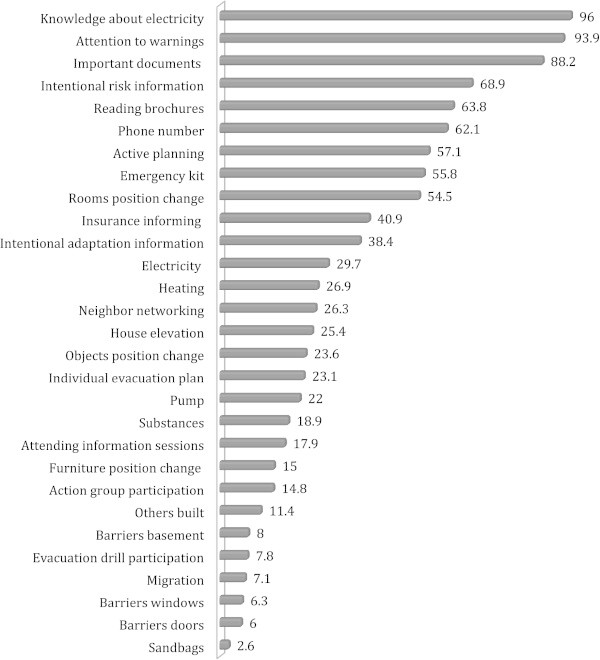
**Proportions of households who undertake specific adaptation measures within all sample areas.**

Descriptive analysis of the data shows a diversity of implemented measures (see Figure 1). The three most frequently implemented measures are: knowledge on how to switch off the electrical supply (96%); paying attention to warnings (93.9%); and storing important documents in places where they can be retrieved quickly (88.2%). The three least implemented measures are storing sandbags (2.6%), buying barriers for doors (6%) and windows (6.3%). Overall, measures that involve low effort and costs for their implementation prevail in contrast to measures of high effort and costs.

### Can different categories of households applying similar combinations of adaptive measures be identified?

Based on the results of the cluster analysis we identified four clusters. Respondents grouped in cluster one constitute the most active group in adaptation. The group makes up 21.4% of the entire sample. In this group, all types of adaptation measures are implemented; often the majority of respondents undertake adaptation measures. For example, 13 measures were implemented by more than half of the respondents of cluster one and eight further measures were implemented by over 30 percent of those respondents. As the largest proportion of this household type implements a broad range of different measures, we termed this first group *the comprehensive*.

The largest group of respondents (38.38%) can be classified as belonging to the second cluster. The dwellers of this group concentrate on non-structural, low-effort measures. On the other hand, they do not often invest in structural measures. Most of the non-structural measures are implemented by more than 50% of cluster two members. The only two measures of which the proportion of cluster two members implementing a measure is greater than the proportion of members within the other clusters are participation in action groups and attending evacuation drills. As cluster two members concentrate on implementing non-structural measures, we termed this category ***the theoreticians***.

A third group, consisting of 26.57% of the respondents, is less active in adaptation. The only three measures which are implemented by a great majority (>70%) of the group members seem to be the basics of being prepared, namely paying attention to warnings, acquiring knowledge on how to switch off the electrical supply and storing important documents in places from which they can be retrieved quickly. Furthermore, they have the lowest proportion of group members implementing structural measures compared to the proportions of members of the other clusters. Households belonging to this category were termed ***the minimalists***.

The remaining 13.65% of the respondents constitute the smallest group of dwellers, who are more active in implementing structural, high-effort measures, compared to the other groups. The group is more active in storing phone numbers and emergency kits as well as important documents and has more often flood-safe electrical appliances and heating; they are also more likely to have barriers for windows and doors and to have moved houses in the past because of being at flood risk. Households belonging to this fourth category were termed ***the structurals***.

### Can differences in allocation of household categories in the three countries be identified?

We explored how similar or different dwellers from the different study sites are in terms of their adaptation behaviour. For this purpose, three Χ^2^ tests per adaptation measure were carried out. First, a global Χ^2^ test showed that the implementation of 19 of a total of 28 adaptation measures (excluding basement barriers) differs significantly between respondents belonging to the three samples. In a second step we tested for similarities and differences of the Danish and German samples in their adaptation behaviour. The results (Table 2) show that the two samples are similar apart from the implementation of seven adaptation measures. German households inform themselves about adaptation, prepare response plans, store important documents in places where they can be retrieved quickly, change the position of objects and substances and store a water pump significantly more often than households of the Danish sample; Danish dwellers, on the other hand, pay attention to flood warnings significantly more often. In a third step, we treated Danish and German respondents as one sample and tested the remaining 13 adaptation measures for significant differences in implementation between the Argentinean and the European samples. The proportion of Argentinean dwellers implementing adaptation measures differs significantly from the German and Danish in thirteen adaptation measures. Dwellers located in Denmark and Germany familiarise themselves significantly more often with the risk of flooding as well as possibilities of insurance. They read brochures, attend information events, and take part in action groups. They also avoid damage from flooding by changing room positions significantly more often than Argentinean dwellers. In contrast, Argentinean dwellers store emergency kits and phone numbers of relevant institutions significantly more often. They further construct flood safe heating and energy supplies significantly more often than dwellers from the European study sites. They also construct barriers for doors and windows in significantly higher numbers. Argentinean households state that they have migrated because of being at flood risk significantly more often compared to households of the European sampling sites.

**Table 2:** **Comparison of adaptation behaviour between countries**

No	Measure	Germany	Denmark	Argentina	European (cum)	1. Global Chi ^2^, df, p	2. Chi ^2^, df, p (Germany – Denmark)	3. Chi ^2^, df, p (European – Argentina)
1	Intentional risk information	84	79	36	81	**67.9;2;.00**	0.9;1;.35	**67.3;1;.00**
2	Intentional adaptation information	52	38	20	45	**23.1;2;.00**	**5.5;1;.01**	**17.4;1;.00**
3	Reading brochures	73	71	43	72	**26.1;2;.00**	0,2;1;.65	**25.9;1;.00**
4	Attention to warnings	93	98	90	96	**7.9;2;.02**	**4.5;1;.03**	**4.6;1;.03**
5	Knowledge about electricity	95	98	95	97	1.4;2:.49	1.0;1;.31	.5;1;.49
6	Attending information events	22	25	3	24	**19.2;2;.00**	0.3;1;.61	**18.9;1;.00**
7	Insurance informing	47	57	12	52	**48.8;2;.00**	2.5;1;.12	**46.3;1;.00**
8	Action group participation	14	24	4	19	**16.9;2;.00**	3.9;1;.05	**12.2;1;.00**
9	Evacuation drill participation	10	7	6	8	0.9;2;.62	0,4;1;.52	.5;1;.48
10	Neighbor networking	31	23	26	27	2.2;2;.34	2.1;1;.15	.05;1;.83
11	Active planning	77	60	27	69	**57.7;2;.00**	**8.6;1;.00**	**50.2;1;.00**
12	Individual evacuation plan	24	17	29	21	4.5;2;.11	1.8;1;.18	2.8;1;.09
13	Phone number	54	49	93	51	**54.5;2;.00**	0.6;1;.45	**53.9;1;.00**
14	Emergency kit	46	53	68	50	**11.1;2;.00**	1.3;1;.26	**9.8;1;.00**
15	Important documents	93	81	92	87	**11.6;2;.00**	**8.7;1;.00**	2;1;.16
16	Rooms position change	68	57	35	62	**23.6;2;.00**	3.1;1;.07	**20.5;1;.00**
17	Furniture position change	20	14	11	17	3.7;2;.16	1.8;1;.18	1.7;1;.19
18	Objects position change	36	20	13	28	**18.1;2;.00**	**8.4;1;.00**	**8.8;1;.00**
19	Substances	20	10	29	15	**12.5;2;.00**	**4.9;1;.03**	**8.4;1;.00**
20	Sandbags	1	4	3	2	2.7;2;.26	2.8;1;.09	.1;1;.71
21	Barriers basement	6	10	Not available	8		1.4;1;.24	-
22	Barriers doors	3	5	11	4	**6.9;2;.03**	0.5;1;49	**6.6;1;.01**
23	Barriers windows	4	6	10	5	4.0;2;.13	0.4;1;.54	**3.8;1;.05**
24	Pump	36	13	16	24	**23.1;2;.00**	**18.9;1;.00**	2.9;1;.09
2325	House elevation	24	30	21	27	2.7;2;.26	1.5;1;.23	1.1,1;.28
26	Electricity	15	17	64	16	**76.6;2;.00**	0.1;1;.82	**76.5;1;.00**
27	Heating	22	19	44	20	**20.4;2;.00**	3.2;1;.57	**20.1;1;.00**
28	Others built	10	9	17	9	3.4;2;.18	0,1;1;.79	3.4;1;.06
29	Migration	5	3	16	4	**14.3;2;.00**	0.4;1;.52	1**4.1;1;.00**

Percentage of households within German, Danish, Argentinean and European (including German and Danish samples) sample areas who undertake single measures and Chi^2^ test values (significant values in bold letters).

Implementation of the remaining adaptation measures that were addressed in the questionnaire did not differ significantly between the European and the Argentinean samples, indicating that the implementation of further adaptation measures in the three sample areas is similar. Most respondents from all three study sites know where to switch off the electrical supply in the house. The quantity of respondents networking with neighbours is similar between study areas: In each country about one third of respondents is engaged in this social process. The same applies to raising the building to avoid getting flooded. Finally, there are no significant differences in the implementation of the following measures, which are applied by a minority of respondents: participation in evacuation drills, changing position of furniture, storing sandbags and other structural measures.

Group affiliations differ geographically; we found differences, e.g. between the Argentinean study sites on the one hand and Danish and German study sites on the other. Nearly half of the Argentinean dwellers (47.9%) can be classified as belonging to the group of *structurals*, while an affiliation to the *minimalists* is also common (31%). As opposed to this, almost half of dwellers from the European countries (49.5%) belong to *the theoreticians*, whereas an affiliation to the *structurals* is rare (1.5%). The *minimalist* (25%) and *the comprehensive* categories (24%) are quite balanced in the European countries. According to the Χ^2^ test, the differences in group affiliation between the Argentinean study sites and the European study sites are significant, indicating that group membership is likely to be related to geographic location. Group affiliations within study sites are shown in Table 3.

**Table 3:** **Composition of types in the sample areas in South America and Europe**

	Clusters			
	The comprehensive	The theoretician	The minimalist	The structural
South American sample areas	14,1	7	31	47.9
European sample areas	24	49.5	25	1,5
Total	21.4	38.4	26.6	13.7

Percentage of dwellers classified into four clusters within South American and European sample areas. A X² test was performed and significant differences in group affiliation between South American study sites and European study sites were found, X² (3, N = 271) = 110,31, p < .001.

### Can differences in behaviour be explained through differences in temporal characteristics?

To analyse whether the identified four categories of households represent different temporal phases of adaptive behaviour, we examined whether they are related to time-dependent variables, i.e. the age of residents and the length of residence in a house and at the coast. A relationship would implicate qualitative changes in adaptive behaviour over time.

With increasing age of residents (up to the age of 69 years), households implement more adaptive measures. When relating the information stemming from the cluster analysis to the age distribution of the sample, differences in adaptive behaviour of different age groups becomes evident. 75% of the youngest group aged 20 to 29 belongs to *the minimalists*. The number of members of all categories increases with age, up to the age of 69 years (with the exception of a decreasing proportion of *the structurals* members in age group 40–49). The differences in adaptive behaviour between age groups are significant (p < .05).

On average, coastal dwellers of the *minimalists* have lived in their house for 13 years (SD = 1.47) and at the coast for 27 years (SD = 2.33). The *comprehensives* are characterized by the longest duration of residence in their house (23 years, SD = 2.29) and at the coast (41 years, SD = 2.75), followed by the *theoreticians* (in the house for 19 years, SD = 1.51; at the coast for 39 years, SD = 3.25). Residents focusing on implementation of structural measures have lived in their house for 12 years on average (SD = 1.81) and at the coast for 20 years (SD = 2.19). As lengths of residence in the house and at the coast were measured on a metric scale, the proportion of explained variance was calculated with η^2^. In both cases, there is a relationship between cluster association and length of residence (η^2^_house_ = .255; η^2^_coast_ = .361). However, values of η^2^ do not indicate, whether there is a positive or a negative direction of the relationship. Table 4 shows the cluster association within age groups.

**Table 4:** **Cluster association of respondents within age groups in percent**

		Clusters				Total
		The comprehensive	The theoretician	The minimalist	The structural	
20-29 years	*	0	12.5	75	12.5	100
	**	0	1	10.2	5.6	3.5
30-39 years	*	17.4	39.1	21.7	21.7	100
	**	7.5	9.1	8.5	27.8	10
40-49 years	*	17.6	47.1	32.4	2.9	100
	**	11.3	16.2	18.6	5.6	14.8
50-59 years	*	23.3	40	28.3	8.3	100
	**	26.4	24.2	28.8	27.8	26.2
60-69 years	*	22.2	45.8	23.6	8.3	100
	**	30.2	33.3	28.8	33.3	31.4
>70 years	*	40.6	50	9.4	0	100
	**	24.5	16.2	5.1	0	14
Total	*	23.1	43.2	25.8	7.9	100
	**	100	100	100	100	100

Note: *Proportions within age groups. **Proportions within cluster membership.

## Discussion

The grouping of household-level adaptation behaviour into four categories gives some indication on the types of policy instruments to use in order to motivate household-level adaptation in coastal areas. In order to provide incentives for the implementation of structural measures, for example, such as storing pumps, elevating the house, constructing flood safe electricity supplies and heating systems as well as storing barriers, communication instruments have to specifically address the groups of the *theoreticians* and the *minimalists*, which make up more than half of the respondents of this study.

The international differences in adaptation policies described above may affect public awareness and perception of flood risk and, finally, adaptation behaviour. By comparing adaptation behaviour in Denmark, Argentina and Germany, significant differences in the number of respondents belonging to certain behavioural categories can be found. In our sample, Danish and German dwellers seem to be more informed and mentally prepared, whereas Argentinean respondents more often take specific structural measures. This result leads us to pose the question, which behaviour indicates a higher adaptive capacity.

We suggest the following alternative explanations for the differences encountered between the study areas: First, adaptation behaviour may depend on the social and institutional background. Second, the provision of risk information is likely to trigger a focus on relevant adaptation measures. Third, dealing with risks and local knowledge can vary between countries and therefore influence behaviour. Finally, adaptive behaviour can depend on the existence of and reliance on public adaptation. However, other implemented adaptation measures did not significantly differ between countries, indicating that an implementation of these measures does not depend on background, risk information, general dealing with risks or public adaptation.

Our results indicate household-level adaptation to be a process where stages of behaviour tend to follow a certain temporal sequence; during the first stage coastal dwellers tend to be minimalists. During the second stage, households shift to become theoreticians and in the third stage they become part of the comprehensive category. An exception pose those households that focus on the implementation of structural measures, whose decision to adapt does not appear to be related to age of occupants or duration of residence at the coast. Future research should focus on this category, as *the structurals* are, in addition to *the comprehensives,* undertaking structural measures, which can be more effective in preventing tangible damage than adaptation measures, which require low costs and efforts.

When interpreting the results of this study, it is important to note that the younger age groups are under-represented in our sample, as 68% of the respondents were over 50 years old. This under-representation most likely results from the fact that ownership of commonhold, and therefore an economic interest in adaptation, is more common at an older age. An indication for this argument is the small share of respondents with children living in the household (27%) and the high share of home owners (83%) in this survey. Furthermore, we noticed that some measures were only relevant for households, which currently have the means to undertake them. For example, moving expensive furniture to upper floors can be only realized if there is an upper floor. Cellars do not commonly exist at the Buenos Aires coast, thus the measure of barriers for basements were not included in the cluster analysis. Although we treated Danish and German respondents as one coherent group, they differed significantly in implementing seven measures. Furthermore, the sizes of the single samples differed; the Argentinean sample is smaller (29%) than the Danish (36%) and German (36%) samples.

Finally, common household adaptation measures should be valued according to their economic benefits. The reduction of costs depends on the type of adaptive measure; for example, temporary safety measures, such as door guards, reduce damage costs by 50% (Thurston et al. 2008). However, in this study, comparable adaptation measures (e.g. protective barriers) were found to be underrepresented in their implementation.

The proposed classification of household-level adaptation styles has potential both for practical applications and future research. In order to inform coastal dwellers about affordable and suitable adaptation, communication should target all categories of households. A typology of household-level adaptation can further assist in addressing specific target groups. For instance, risk communication through social media could make use of the classification and address younger age groups that often belong to the category of minimalists. Furthermore, by identifying the factors which have an effect on decisions concerning adaptation, we improve our understanding of how behavioural changes and investment decisions can be encouraged. The proposed typology can provide indications about unexplored drivers of household-level adaptation for future research (e.g. by further focusing on the characteristics of specific categories of households) and improve our understanding of existing patterns in household-level adaptation. The typological construction can also help to manage larger data sets containing information about household-level adaptation.

## Conclusions

This paper proposes a typology of coastal households with regard to their adaptive behaviour. Based on questionnaire surveys carried out in coastal regions in Germany, Denmark and Argentina we have identified four categories of coastal households, which differ in their adaptive behaviour: the theoreticians, the minimalists, the structurals and the comprehensives. In most of the cases the respondents of this study belonged to the behavioural type which we termed *the theoreticians*, signalling interest in being informed, rather than taking structural measures. By analysing category affiliation geographically, further patterns emerged. Belonging to a specific behavioural type appears to depend on external characteristics, such as differences in the institutional background, in local knowledge or the general existence of public measures. Our findings show that household-level adaptation is, to a large extent, a process that develops over time. Coastal dwellers start with basic measures, then prepare themselves mentally and, with progressing age and duration of residence, start implementing more time- and effort-intensive measures. The small group of respondents which we termed *the structurals* constitutes a special case: group affiliation is not related to time. Future research could explore the reasons for concentrating on the implementation of structural measures, as these measures have a large potential in reducing damage.

This study emphasizes the importance of integrating household-level adaptation into coastal risk management, while at the same time considering the diversity of styles of household-level adaptation. We conclude that risk communication can only reach the entire group of households and increase versatile adaptation behaviour by addressing all behavioural types of households. The typology described is potentially applicable for describing behavioural types of coastal households in different areas and also in other environmental risk contexts.
